# Automated and Low Computational Cost Thermo-Mechanical Simulation of Arbitrary GMAW T-Joint Welds Using a Moving Heat Source

**DOI:** 10.3390/ma19051021

**Published:** 2026-03-06

**Authors:** Sebastian Santarrosa-Rodriguez, Israel Martínez-Ramírez, Motomichi Yamamoto, Rocio A. Lizarraga-Morales, Felipe J. Torres, Isaí Espinoza-Torres, Víctor Manuel Vega-Gutierrez

**Affiliations:** 1 Division of Engineering, Irapuato-Salamanca Campus, University of Guanajuato, Salamanca-Valle de Santiago Highway, km 3.5 + 1.8 Palo Blanco Community, Salamanca 36787, Mexico; s.santarrosarodriguez@ugto.mx (S.S.-R.); ra.lizarragamorales@ugto.mx (R.A.L.-M.); fdj.torres@ugto.mx (F.J.T.); vm.vegagutierrez@ugto.mx (V.M.V.-G.); 2 School of Engineering, Hiroshima University, 1-3-2 Kagamiyama, Higashi-Hiroshima 739-8511, Japan; motoyama@hiroshima-u.ac.jp (M.Y.); isai.et@purisima.tecnm.mx (I.E.-T.); 3 Department of Automotive Systems, Tecnológico Nacional de México/ITS de Purísima del Rincón, Blvd. del Valle 2301, Guardarrayas, Purísima del Rincón 36425, Mexico

**Keywords:** welding simulation, efficient finite element model, thermo-mechanical analysis, Goldak double ellipsoidal, heat source, automated macro, gas metal arc welding, parametric, induced distortion

## Abstract

Gas Metal Arc Welding (GMAW) is widely adopted in automated manufacturing industries where the accurate prediction of thermal fields and welding-induced distortions is essential to ensure joint integrity of the parts; however, finite element modeling, as the most reliable non-destructive predictive approach, remains time-consuming and highly user-specialized. This work presents an automated and low computational cost thermo-mechanical finite element methodology implemented in Ansys Parametric Design Language (APDL) for the parametric analysis of GMAW T-joints, integrating automated geometry generation, meshing, heat source implementation, and thermo-mechanical modeling for different beam and weld seam dimensions under continuous or intermittent single-pass configurations. A volume element selection strategy is introduced to limit heat input calculations to the active weld pool region, achieving up to a 50% computational time reduction while maintaining high predictive accuracy, in contrast with conventional and partial selection methods. Overall script performance was validated through temperature and displacement comparisons between the numerical and experimental results of two T-joint configurations using SM490A structural steel specimens. The results demonstrate that the developed macro provides a useful tool for automated thermo-mechanical welding analysis, significantly reducing model preparation effort while enabling the evaluation of parametric T-joint geometries and welding conditions with a low computational cost focus.

## 1. Introduction

Welded T-joints are widely used across multiple industrial sectors due to their versatility in adapting to various geometries and their suitability for automated manufacturing, particularly when using continuous-feed electrodes as in the Gas Metal Arc Welding (GMAW) process, also known as MIG/MAG welding. Given their extensive application and the inherent complexity of the welding phenomenon, understanding the thermal and mechanical behavior of materials during joining is essential to ensuring high weld quality and, therefore, a reliable joint [1,2,3].

In recent years, advancements in computational power have increased the adoption of finite element simulations as a non-destructive and cost-effective alternative to support decision making in welding processes, enabling accurate prediction of the structural behavior from temperature gradients during material fusion. Building upon this growing reliance on finite element simulations, thermo-mechanical modeling of the GMAW process using a moving heat source has become one of the most widely adopted approaches for welding simulations in both academic and industrial contexts, balancing predictive accuracy and improving computational cost efficiency [4,5,6]. This approach has been widely applied to butt-welding configurations with a heat source acting normal to a horizontal plane, and more recent studies extend it to T-joint configurations by incorporating heat source inclination, showing its influence on numerical prediction accuracy [7,8,9].

Despite its broad applicability in industrial and research contexts, reducing computational cost remains one of its main challenges, motivating alternative approaches based on material and physical model simplification. One of the earliest and most widely adopted strategies for improving numerical efficiency in welding simulations consists of minimizing nonlinearities by considering temperature-dependent material properties as temperature-independent. This simplification has been applied across various welding processes, as illustrated by the work of Komen et al. [10] in Submerged Arc Welding (SAW), Shuai et al. [11] in LASER-welded T-joints, and Olivier et al. [12] in Metal Inert Gas Welding (MIG). Even though this simplification reduces the simulation time, thermal and structural results may exhibit variations, since both the thermal field and the resulting mechanical response show strong sensitivity to the temperature dependence of material properties [13,14]. Arthur et al. [15] demonstrated that assuming constant properties leads to inaccurate thermal predictions when contrasted with models incorporating nonlinear material behavior in laser welding simulations. His findings underscore that, for reliable temperature estimation—and consequently, for accurate prediction of distortions—temperature-dependent properties must be used in welding analyses.

Beyond material modeling considerations, several strategies aimed at improving numerical accuracy while maintaining low computational costs have been reported in the literature, particularly through the development of advanced heat source formulations, scripting methodologies and mesh optimization techniques. This has been demonstrated in several studies, such as the work by Guoxiang et al. [16], who employed a coordinate-transformation formulation to capture heat-source rotation in T-joint welding simulations, complemented by a non-uniform mesh to mitigate computational time. Similarly Zhifeng et al. [17] proposed a combined heat source model—integrating a double ellipsoidal and a cylindrical component—to better represent fusion zone (FZ) morphology, while implementing a pyramidal-to-hexahedral hybrid mesh to reduce computational demand in butt welding simulations.

On the other hand, the industrial applicability of thermo-mechanical welding models faces an additional challenge related to the lack of low-intervention and workflow-efficient modeling methodologies, resulting in a strong dependence on expert knowledge and time-consuming model preparation. Consequently, recent research has increasingly focused on welding automation, optimization, and multi-case analysis, aiming to improve adaptability, ease of implementation, and generalization across different welding scenarios. This trend is reflected in the work of Zhifeng Li et al. [17], who employed a backpropagation neural network (BPNN) combined with a genetic algorithm (GA) to predict weld bead geometry and hardness from process parameters, enabling the optimization of welding conditions. Similarly, a more generalized and transferable approach can be found in the work of Hao Jiang et al. [18], who developed a heat source parameter identification network (HSPINet) based on a residual convolutional neural network (ResNet) to automatically identify heat source parameters across varying process conditions and different joint geometries, significantly enhancing the efficiency and robustness of heat source parameter identification, and more recently, Fernández-Zabalza et al. [19], who proposed a deep learning-based real-time monitoring framework for wire arc additive manufacturing (WAAM), enabling seamless integration into industrial control systems reliable diagnostics without additional latency, thereby reinforcing the growing emphasis on efficiency and automation in welding process analysis and control. Despite the extensive body of research on welding simulation—including advanced heat source formulations, solution time reduction strategies, and scripting-based implementations to automate specific stages of the analysis [20,21,22,23,24]—the literature lacks a volume-based element selection strategy for computational time reduction that is integrated in a fully parametric framework capable of generating the complete end-to-end welding analysis, from volumetric geometry and mesh generation to heat source application and coupled thermo-mechanical analysis, based solely on a reduced set of input parameters.

Accordingly, this study addresses these limitations by introducing an APDL macro, which is supported by Fortran routines to generate models for the thermo-mechanical analysis of a GMAW process in parametric T-joint configurations, and wide range of flange and web geometries; welding conditions, including voltage, current, and welding speed; base-metal materials defined through their associated mechanical and thermal properties; fillet weld geometries automatically determined from their characteristic dimensions; multiple double-ellipsoid heat source parameter combinations; and, in the current implementation, several single continuous or intermittent weld seam distributions, defined through preprogrammed variables accessed via pop-up windows and a pseudo-algorithm that enforces specific structural constraints associated with physical fixture positioning, using temperature-dependent material properties with a low solution time focused methodology by an efficient scripted volume element selection strategy and model simplifications, thereby reducing computational cost without compromising accuracy.

The distinction between the proposed methodology and most closed literature [16,25,26,27,28,29,30,31] is summarized in Table 1, highlighting that, unlike prior models requiring extensive manual adjustments, the proposed framework reduces preprocessing complexity and simulation cost by integrating a novel volume-based element selection into a parametric end-to-end analysis, allowing faster solution times.

Experiments were conducted on JIS-SM490A structural steel, and the resulting temperature distribution and induced deformation patterns were evaluated and compared through both numerical and experimental measurements, showing good agreement, with a maximum peak temperature and thermal deformation deviation of 25 °C and 0.28 mm respectively. Together, these results and the proposed automation strategy provide a reusable platform for efficient numerical GMAW process analysis, reducing finite element model preparation time and user intervention while enabling systematic evaluation of thermo-mechanical responses across parametric T-joint configurations and user-defined welding conditions.

## 2. Materials and Methods

### 2.1. Algorithmic Approach

**Figure 1:**
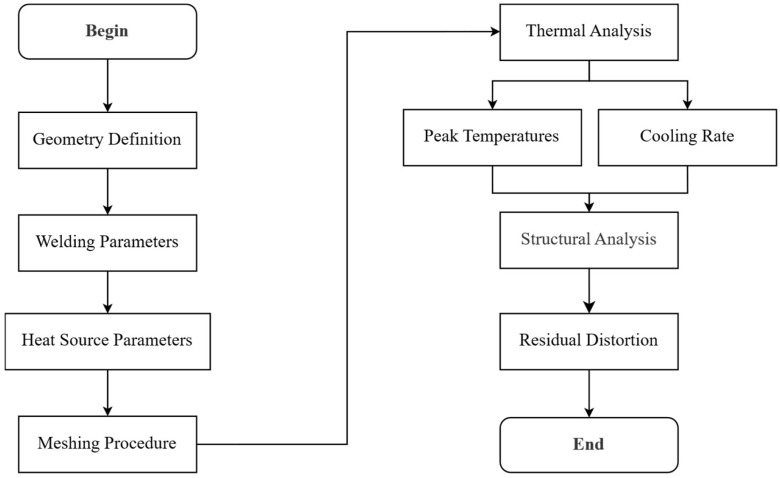
Sequential macro workflow for parametric GMAW process analysis in APDL.

A macro is a powerful tool in APDL encapsulated in a file with the .MAC extension, which, by simply invoking it through the command window within the ANSYS 2025 mechanical user interface, enables the execution of parameterized analyses in a fully automated manner. Its greatest advantage lies in the ability to streamline repetitive tasks, reduce human error, and accelerate the simulation workflow. Instead of manually redefining boundary conditions, material properties, or geometric parameters for each case, the macro allows us to predefine these variables and run complex sequences of analyses with a single command [32]. Figure 1 shows the flow chart of the overall T-joint welding simulation macro, which is organized into three main stages: model generation, thermal analysis, and structural analysis.

In the first stage, geometric configuration of the welded joint and welding parameters are defined, including the heat source model and mesh. The thermal analysis then simulates the welding process, providing the temperature evolution and cooling behavior. Finally, the structural analysis employs the thermal field as input to predict residual distortions and associated stress.

#### 2.1.1. Geometry Definition and Welding Condition

**Figure 2:**
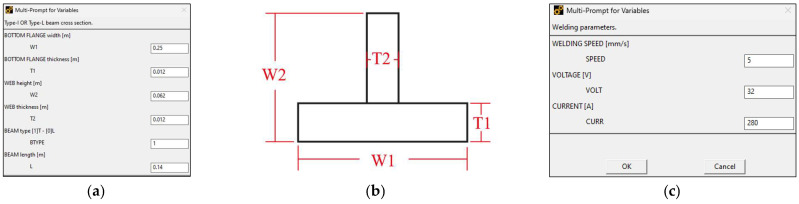
Initial pop-up windows preprogrammed for input parameter definition: (**a**) geometric definition of the base material dimensions; (**b**) geometry configuration and notation; (**c**) welding conditions.

Upon execution of the macro in a new APDL environment, the input stage begins through a graphical user interface that allows the user to define the required parameters for the analysis. The geometric configuration of the base material is specified by four parameters representing the flange width (W1) and flange thickness (T1), as well as the web width (W2) and web thickness (T2), as illustrated in Figure 2a, with the corresponding beam dimension symbology shown in Figure 2b. Similarly, user-defined inputs are required to specify the welding parameters, including welding speed (SPEED), working voltage (VOLTAGE), and welding current (CURRENT), as shown in Figure 2c. All parameters are provided through a preprogrammed pop-up interface implemented within APDL.

**Figure 3:**
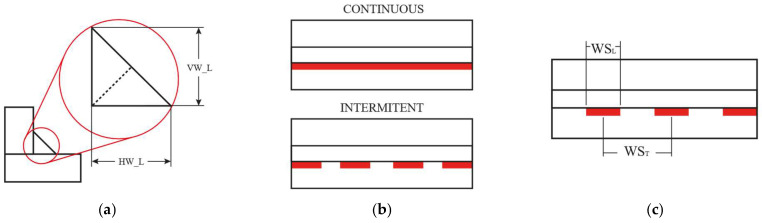
Weld seam user definition: (**a**) weld seam symbolic notation used in this study; (**b**) graphic representation of the weld seam location on the beam; (**c**) intermittent weld seam layout parameters.

The subsequent stage involves specifying the welding conditions, which comprise the fillet weld bead geometry and layout. At this stage, the weld bead geometry is defined using two weld size parameters: the horizontal fillet weld leg length (HW_L) and the vertical fillet weld leg length (VW_L), as illustrated in Figure 3a. Following the definition of the weld bead geometry, the deposition pattern is specified by defining whether the analysis corresponds to a single continuous or intermittent pass, as shown in Figure 3b. For the intermittent single pass condition, the weld layout is further defined by the weld seam length (WS_L_) and pitch (WS_T_), as illustrated in Figure 3c.

The finite element formulation considers three primary assumptions: (i) the influence of joint fixturing; (ii) the thermophysical properties of the material; and (iii) the process efficiency, which is treated as an approximate parameter and refined through iterative calibration to achieve agreement with the thermocouple temperature histories.

According to the primary assumptions adopted in this work, a physical simplification was introduced by neglecting contact elements. Instead, a volume segmentation strategy was implemented to ensure accuracy and computational efficiency. The main limitation of this approach is the inability to simulate small separations between the flange and the web of the beam. However, this limitation does not have a significant impact on the prediction of global deformations [33].

**Figure 4:**
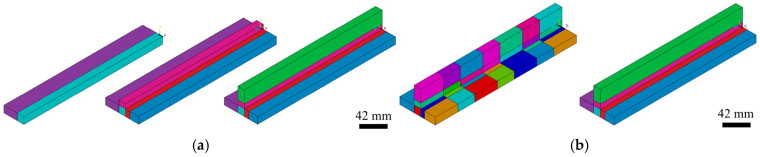
Base material volume generation: (**a**) sequential construction representation; (**b**) volume segmentation with an intermittent (**left**) and a continuous weld bead (**right**).

Consequently, following the macro workflow, the volumes corresponding to each part of the beam are generated independently and subsequently merged by overlapping keypoints (KP) and lines (L), as shown in Figure 4a. The partitioning of the full beam volume into sub volumes is closely linked to the weld layout—for example, whether intermittent or continuous weld beads are defined, as shown in Figure 4b—and enables the use of a structured (mapped) mesh in the later meshing stage.

**Figure 5:**
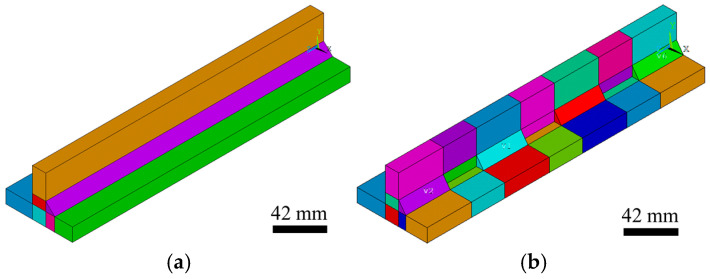
Weld seam volume definition used in the finite element model: (**a**) continuous weld seam volume distribution; (**b**) intermittent weld seam volume distribution.

The volume generation process concludes with the creation of the weld bead volume directly over the previously defined base material volume, followed by the required cutting operations according to the specified weld bead layout. Whether a continuous weld configuration is considered (Figure 5a), in which the formulation supports cases where the weld bead extends over the full beam length as well as configurations with gaps near the beam ends, or an intermittent weld configuration, in which the base material is segmented according to the intermittent weld seam definition (Figure 5b), the alignment of the base material and weld seam geometries through predefined segmentation allows all volumes to be merged using the same technique previously applied, by overlapping the newly generated keypoints (KP) and lines (L) of the weld seam with those of the base material to obtain a single, consistent model.

For this geometry generation process, the same material (i.e., same thermal and mechanical properties) was assigned to both the base material and the weld bead, to reduce model complexity and computational cost. Under this assumption, the model is expected to accurately capture overall temperature gradients and weld-induced strains, while local effects associated with microstructural variations in the weld metal are not explicitly resolved [16].

#### 2.1.2. Heat Source Model Definition

**Figure 6:**
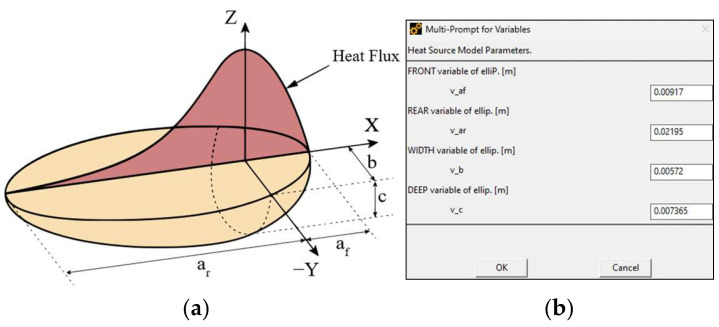
Goldak’s double-ellipsoid heat source model: (**a**) Schematic representation of the volumetric heat density distribution; (**b**) Definition of heat source parameters through preprogrammed user input window.

Among the various formulations used to represent heat input in welding simulations, the volumetric double ellipsoid heat source model, commonly referred to as Goldak’s double ellipsoidal model, Figure 6a, is considered the most suitable approximation for describing the thermal behavior of the GMAW process as it overcomes the limitations in capturing the asymmetric temperature gradients ahead of the arc and in the trailing region of the molten pool [21,34,35]. Accordingly, the parameters of the double ellipsoidal heat source are defined through a preprogrammed pop-up interface as shown in Figure 6b.

The applied heat source governs the spatial distribution of thermal flux within the weld pool by representing it as two ellipsoidal regions-oriented perpendicular to each other, enabling independent specification of the heat source dimensions in the plane orthogonal to the welding direction. The associated heat input is defined analytically through established mathematical expressions. The corresponding equations are presented below:

(1) $$q_{f}\left(x,y,z\right)=\frac{6\sqrt{3}f_{f}Q}{a_{f}bc\pi \sqrt{\pi }}\;\cdot e^{\left(-\frac{\beta {\left[x+\nu \left(\tau -t\right)\right]}^{2}}{a_{f}^{2}}\right)}\cdot e^{\left(-\frac{\beta y^{2}}{b^{2}}\right)}\cdot e^{\left(-\frac{\beta z^{2}}{c^{2}}\right)}$$

(2) $$q_{r}\left(x,y,z\right)=\frac{6\sqrt{3}f_{r}Q}{a_{r}bc\pi \sqrt{\pi }}\cdot e^{\left(-\frac{\beta {\left[x+\nu \left(\tau -t\right)\right]}^{2}}{a_{r}^{2}}\right)}\cdot e^{\left(-\frac{\beta y^{2}}{b^{2}}\right)}\cdot e^{\left(-\frac{\beta z^{2}}{c^{2}}\right)}$$

where $a_{r}$ represent the major rear semiaxis, $a_{f}$ represent the major front semiaxis, b and c represent the semiaxes of the ellipsoids, x, y and z represent space coordinates of the heat source, τ is a lag factor of time t, β is a dimensionless heat source constant (commonly taken as 3 in the literature, corresponding to a power density that decreases to 0.05 of its maximum value at the surface of the ellipsoid) and Q represents the total input rate, which is established according to the welding parameters voltage V, current I and process efficiency η. The fraction of deposited heat on the front and rear quadrant of the domain is defined by $f_{f}$ and $f_{r}$ and must satisfy the continuity condition $f_{f}+f_{r}=2$ [36].

#### 2.1.3. Meshing

Two types of eight-node hexahedral elements were employed: SOLID70 for thermal analysis, and its analog element, SOLID185 for mechanical analysis. The main difference between both elements lies in the application domain, while the SOLID70 element is formulated for a 3-D, steady-state or transient thermal analysis, its use is limited to a single nodal degree of freedom corresponding to temperature, consequently, when structural behavior is also of interest, the thermal element must be replaced by an equivalent structural formulation. In this study, SOLID185 was adopted, as it preserves the same eight-node topology while incorporating three translational degrees of freedom at each node in the *x*, *y*, and *z* directions.

Due to the nature of the phenomena involved, grid size and mesh design have a significant influence on both the thermal analysis and the subsequent structural response of the model. Accordingly, the mesh generation algorithm was designed based on physical relevance, numerical accuracy, and computational efficiency, and is controlled by three primary parameters: partition factor ($F_{DIV}$), normalization factor ($F_{REF}$), and longitudinal scaling factor ($F_{LEN}$), allowing for easy and fast adjustment to different model geometry configurations.

**Figure 7:**
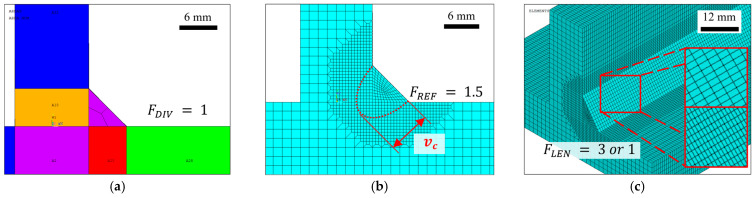
Meshing strategy and element size controls applied in the finite element model: (**a**) elements in weld seam cross section area; (**b**) penetration selection refinement; (**c**) extruded element lengths, using ratios of three (top) and one (bottom).

Mesh consistency was first ensured by generating an initial 2D mesh through the mesh-only MESH200 elements to keep a uniform discretization over the entire beam cross-section derived from the weld bead mesh. The parameter $F_{DIV}$ defines the number of cuts along the edges of the fillet weld profile, as illustrated in Figure 7a. The resulting smallest element size at the weld toe was subsequently adopted to mesh the remain cross section areas.

Since the weld pool constitutes the primary zone of interest, where high temperature gradients are present, a refined mesh in this location is required to accurately capture the fusion zone. Therefore, in the subsequent step, local mesh refinement is applied in the vicinity of the weld seam to enhance solution accuracy. This refinement is achieved through a circular selection centered at the midpoint of the weld face line by ($F_{REF}$), which controls the extent of element refinement relative to the weld penetration depth $v_{c}$ of the heat source model, as shown in Figure 7b. A resulting ratio between coarse and refined mesh regions equal to 1.5 was selected such that further refinement produced negligible variations in the predicted temperature field, particularly in the weld pool region, as well as in the displacement results away from the weld pool, while maintaining a reduced computational cost for the specimen configurations evaluated in this study.

Finally, a three-dimensional mesh is generated through an area-mesh extrusion process along the welding direction, where ($F_{LEN}$) governs elements extrusion size by using the previously refined weld bead element size as a reference value, as illustrated in Figure 7c.

The default values of the three parameters of meshing implemented in the macro, ($F_{DIV}$) of 3, ($F_{LEN}$) of 1.5 and ($F_{REF}$) of 1.5, were defined through an iterative process supported by a mesh convergence analysis. These parameters may provide a well-established starting point for a wide range of T-joint and weld seam geometrical configurations (i.e., different flange and web lengths, and thicknesses, as well as weld toe dimensions). However, as shown in the following sections, more precise control for specific study cases can be readily achieved by adjusting these three parameters.

### 2.2. Thermal Analysis

The thermal analysis consists of solving the heat conduction equation—Fourier’s second law for isotropic materials—over the entire model domain. Based on the principle of energy conservation for a homogeneous, continuous, and isotropic medium, the governing heat conduction equation can be expressed as

(3) $$\rho c_{p}\frac{\partial T}{\partial t}={k\nabla }^{2}T+\dot{Q}$$

where T denotes the temperature, t is time, ρ is density of conducting medium, $c_{p}$ is the specific heat capacity of the medium, k is the material thermal conductivity, and $\dot{Q}$ represents the volumetric heat generation rate.

The solution of the governing equation, Equation (3), depends on the initial and boundary conditions applied to the domain under analysis. The initial condition is defined as the temperature of the base material at the beginning of the welding process, $T_{0}$. This temperature corresponds to the ambient temperature of the working environment; however, in welding processes involving material preheating, the initial temperature is set equal to the preheating temperature, as expressed in Equation (4):

(4) $$T(x,y,z)=T_{0}$$

Heat losses due to convection at the material surface are described by Newton’s law of cooling, which defines the convective boundary condition as

(5) $$q=h_{c}(T-T_{\infty })$$

where q is the heat flux lost by convection, $h_{c}$ is the convective heat transfer coefficient, T is the surface temperature of the body, and $T_{\infty }$ denotes the room temperature of the surrounding environment.

#### 2.2.1. Heating and Cooling Cycle

Based on the previously established formulation, the complete thermal analysis was divided into two stages: the heating cycle and the cooling cycle.

Once the heat source has traversed the entire weld seam and all corresponding load steps have been saved (i.e., completion of the heating cycle), the cooling cycle is initiated by deactivating the heat source and solving the subsequent heat dissipation through conduction and convection mechanisms. The cooling analysis begins from the temperature field obtained at the final heating load step and is carried out over a user-defined time interval using predefined film coefficients and ambient temperature conditions, thereby capturing the material thermal response as a function of the resulting cooling rate [37].

For the numerical implementation of this formulation, the thermal analysis was configured using an implicit backward Euler time integration scheme combined with a ramped thermal load step. The heating stage was discretized into five sub-steps, which were iteratively adjusted according to convergence criteria to ensure numerical stability and accuracy. At each sub-step, equilibrium was enforced using a full Newton–Raphson solution procedure to guarantee convergence of the nonlinear thermal problem arising from temperature-dependent material properties and moving heat source effects. For the cooling stage, a single load step was employed, taking advantage of the reduced nonlinearities and increased numerical stability associated with decreasing temperatures [38], while the number of substeps is calculated from the ratio of cooling time and time increment.

In line with the focus on low computational cost and the corresponding analytical derivations of temperature fields in welding processes, which typically rely on several simplifying assumptions [39], the thermo-mechanical simulation aimed at predicting welding-induced distortions driven by transient thermal loads adopts the following assumptions. First, isotropic heat conduction is considered. Temperature-dependent material properties of JIS-SM490A were applied for both the base and fillet weld materials; therefore, the mechanical formulation is restricted to the solid phase, since the contribution of the molten material to the global displacement response is negligible. This effect is inherently captured through the temperature-dependent reduction of Young’s modulus. Only thermal losses due to conduction within the material and convection with the surroundings are accounted for, while convective heat transport within the molten pool is neglected, as it has a limited influence on the final distortion response [40].

**Figure 8:**
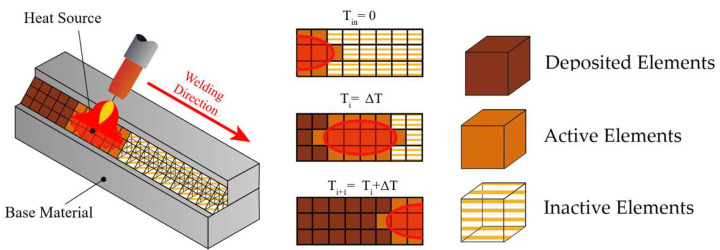
Schematic representation of the material deposition procedure used for the thermal analysis in the macro.

Building on the thermal and mechanical assumptions described above, the melting of the filler metal and the subsequent weld bead formation were modeled using the element birth and death technique [32]. The weld bead volume is represented through three element states: inactive elements (to be deposited), active elements (volumetric heat generation), and deposited elements (previously deposited material). At each load step, the elements located within the heat-source volume are identified, activated, and assigned a heat-generation rate based on their position in the local coordinate system centered at the heat source. As the heat source advances along the weld path, new elements enter the activation zone in the next load step (Δ*LS*), while previously active elements fall outside the heat-source region representing solidified deposited material, as illustrated in Figure 8 [41].

#### 2.2.2. Volume Element Selection Strategy

In previous investigations, heat generation from the double ellipsoidal heat source formulation was evaluated by checking all elements and associated nodes within the entire model domain and assigning a heat input based on their relative position to the heat source origin, with negligible contribution from elements located outside the power density region. Although this approach is straightforward—since no prior element selection is required—it is computationally demanding, as it involves evaluating every node in the model at each load step. Consequently, its performance is strongly dependent on the computational resources available, making it robust but inefficient for large-scale or automated analyses.

To overcome the high computational resources that imply to apply the heat source to all nodes in the finite element model, a volume selection strategy is implemented. In this approach, only the elements located within the double ellipsoidal heat source volume are considered for heat generation assignment, which is calculated according to their position relative to a local coordinate system centered at the heat source origin.

**Figure 9:**
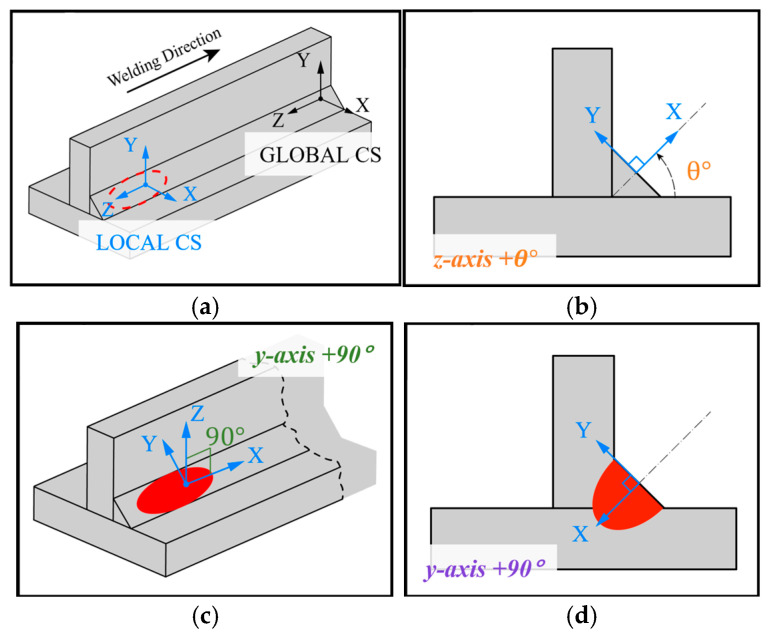
Low computational cost element volume selection strategy for moving heat source application in the thermal analysis, highlighting the reduced element set used for heat generation assignment: (**a**) creation of the local coordinate system; (**b**) first *z*-axis rotation; (**c**) second *y*-axis rotation and first transverse selection; (**d**) third *y*-axis rotation and final longitudinal selection.

The volume selection procedure and the associated element identification are carried out in APDL through the following four steps:

1. A local cylindrical coordinate system is created and positioned at the center of the heat source using the global coordinate system as reference, as shown in Figure 9a;
2. A rotation about the *Z*-axis is applied to align the *Y*-axis with the weld bead face, Figure 9b;
3. A second rotation about the *Y*-axis is then performed to adjust the X–Y plane relative to the weld bead face, allowing the first transverse selection of elements within the ellipsoid projection, Figure 9c;
4. A third rotation about the *Y*-axis aligns the X–Y plane with the bead’s cross-section area, enabling a second longitudinal selection of the elements contained in the ellipsoid projection, Figure 9d.

Since applying the heat generation formulation to elements with negligible contribution (i.e., outside the heat source domain) does not affect the solution results but only increases the model generation time, and excluding elements that should receive heat input due to an overly restrictive selection range may lead to inaccuracies in the predicted temperature field, the programmed range—determined through an iterative procedure as 1.5 times the experimentally measured weld width, length, and penetration depth—was chosen to provide a safety margin, ensuring that all elements potentially influenced by the heat source are included. The same selection criterion was applied at the welding start, stable, and termination regions of the process, accounting for the rapid temperature rise in the base material caused by the moving heat source, which shifts spatially according to a local coordinate system.

### 2.3. Structural Analysis

Following the workflow of the finite element macro, the temperature history obtained from the previous thermal simulation is used as input data for the calculation of the resulting deformations during and after the welding process. This procedure can be carried out using either a sequential strategy or a direct coupling strategy. In the sequential approach, each field is solved independently, and the results of the first analysis are transferred as loading conditions to the second. In contrast, the direct method solves the coupled degrees of freedom simultaneously, which typically introduces additional nonlinearities. Due to these nonlinearities and the increased computational cost associated with the direct approach, a sequential solution strategy is implemented in the present work, as it allows each field to be solved independently while avoiding multi-field iterations [38].

The thermal cycle applied to a welded joint induces expansions and contractions that vary both in time and with respect to the location of the heat source. Since these expansions are inherently non-uniform, the material in the hot regions near the weld is constrained by the colder zones located farther away. This constraint leads to plastic deformation and, consequently, to the development of residual stresses that remain in the material after the temperature equilibrates with the surrounding environment [36].

Accordingly, the total strain in the material can be expressed as

(6) $$\varepsilon =\varepsilon_{el}+\varepsilon_{pl}+\varepsilon_{t}$$

where the terms on the right-hand side correspond to elastic, plastic, and thermal strains, respectively.

The mechanical response of the welded material is described assuming an elastic behavior governed by isotropic Hooke’s law, with a Young’s modulus that varies as a function of temperature. Thermal strains are incorporated through the thermal expansion coefficient to account for the deformation induced by temperature variations. On the other hand, the plastic response of the material is modeled using a rate-independent plasticity formulation; in this manner, yielding is defined according to the von Mises criterion, while the post-yield behavior is represented by a bilinear isotropic hardening law, allowing the strain-hardening effects of the welded material to be adequately captured [38].

### 2.4. Validation Experiments

**Figure 10:**
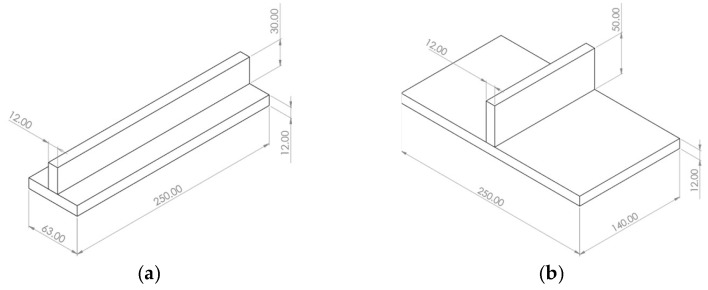
Specimen geometry and dimensions (units in mm) used for the numerical simulations: (**a**) thermal analysis model; (**b**) structural analysis model.

To evaluate the reliability of the automated welding analysis, two experimental specimen configurations were employed: one dedicated to the thermal analysis evaluated through temperature history comparisons, and a structural setup evaluated via displacement measurements. The dimensions of the specimens with a thickness of 12 mm, used for both thermal and structural experimental and numerical simulations, are illustrated in Figure 10a and Figure 10b respectively.

Both the base metal and the stiffener used in the thermal and structural analyses were modeled as JIS-SM490A structural steel. In this GMAW process, a 1.2 mm diameter JIS YGW11 solid wire was used for the experimental welding. However, as explained in previous sections, a mechanical simplification was adopted in the numerical analysis, whereby the filler material was assumed to have the same mechanical properties as the base material. Under this assumption, the chemical composition of both the base and filler materials is summarized in Table 2.

**Figure 11:**
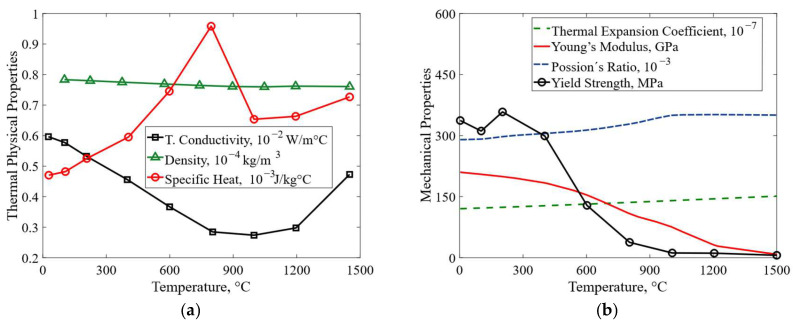
Temperature-dependent properties of SM490A steel: (**a**) thermal; (**b**) mechanical [42].

Accordingly, temperature-dependent thermal and mechanical properties of JIS-SM490A steel, Figure 11a and Figure 11b respectively, were adopted for modeling consistency and simplicity in both the base material and the weld bead. A cut-off temperature of 1500 °C was defined due to the availability of data in the literature. Welding conditions are listed in Table 3.

#### 2.4.1. Temperature Distribution

**Figure 12:**
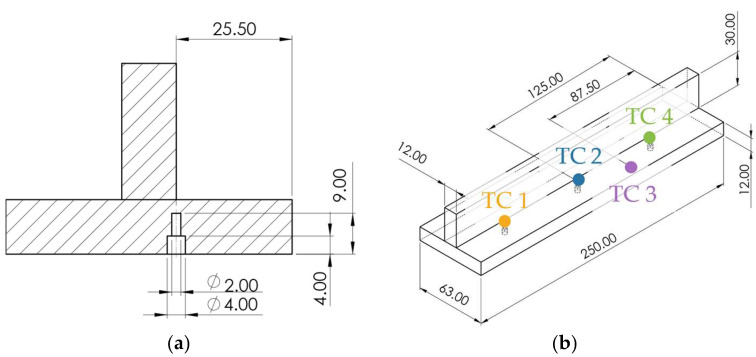
Thermocouple layout and locations in the specimen for thermal analysis (units in mm): (**a**) cross-sectional view showing embedded thermocouple positions; (**b**) isometric view of the thermocouple arrangement.

For temperature measurements near the weld fusion zone, thermocouple one (TC1), thermocouple two (TC2), and thermocouple four (TC4), named in the order of the welding direction, were embedded in the base material using 2 mm-diameter drilled holes, as shown in Figure 12a. An additional thermocouple numbered as three (TC3), was placed on the material surface to record the surface temperature, as illustrated in Figure 12b. All thermocouples used were type-R with a diameter of 0.3 mm.

**Figure 13:**
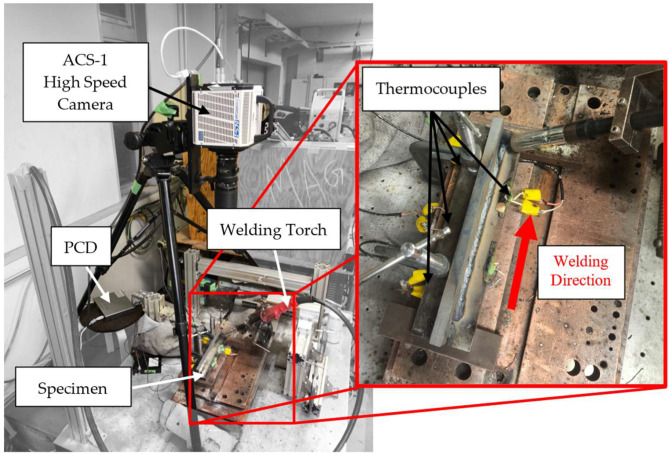
Experimental specimen configuration and instrumentation used for thermal analysis validation.

In addition, a high-speed MEMRECAM ACS-1 camera (nac Image Technology Inc., Tokyo, Japan), was employed to experimentally determine the front and rear longitudinal dimensions of the heat source, denoted as $a_{f}$ (major front semiaxis) and $a_{r}$ (major rear semiaxis), respectively, based on direct observation of the weld pool during the filler material deposition process, as shown in Figure 13.

#### 2.4.2. Displacement Distribution

**Figure 14:**
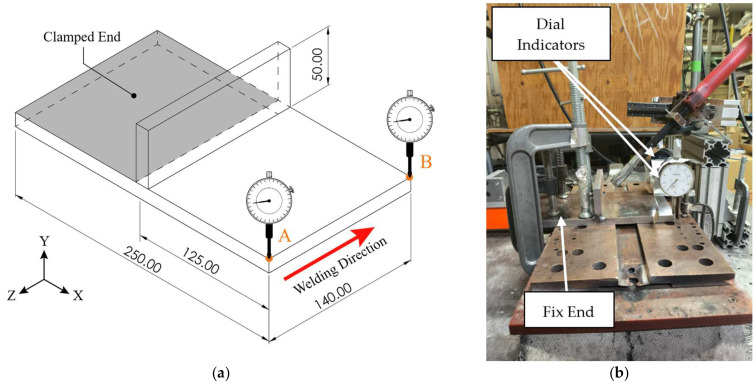
Specimen configuration for structural analysis validation: (**a**) schematic specimen dimensions (units in mm) and dial indicator locations; (**b**) experimental setup used for displacement and angular distortion measurements.

The structural analysis was validated through a comparison between numerically predicted and experimentally measured displacements and angular distortion after welding. The base material was clamped at one end, while the opposite end remained free to deform under the contraction induced by the weld bead. On the other hand, displacement measurements were experimentally recorded at two locations on the unconstrained end using dial indicators positioned at equal distances from the weld bead and distributed along the beam length. Schematic and actual experimental setups are illustrated in Figure 14a and Figure 14b, respectively.

## 3. Results and Discussion

### 3.1. Experimental Study

#### 3.1.1. Thermal Response—Experimental

**Figure 15:**
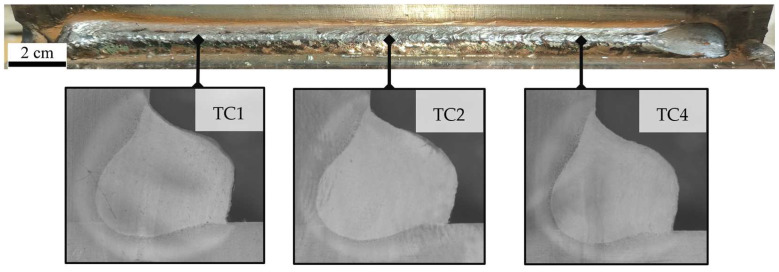
Weld bead appearance and cross-sectional macrographs at the three thermocouple locations used for thermal validation.

For weld seam quality determination, three equally distributed cross-sections along the weld seam length were prepared using a non-heat polishing process and the resulting weld bead exhibited a uniform surface geometry with no macroscopically detectable defects, indicating stable welding conditions and proper filler metal deposition, as shown in Figure 15.

**Figure 16:**
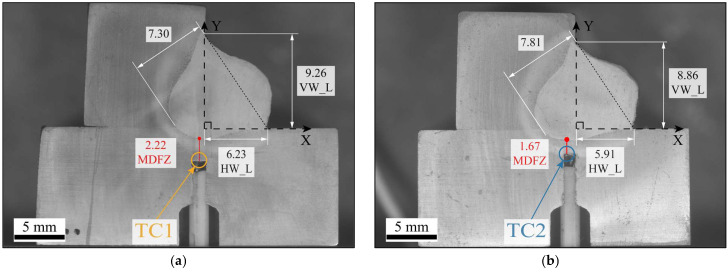

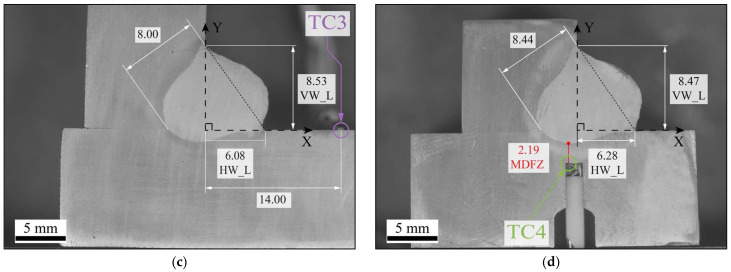
Cross-sectional geometric dimensions of the weld bead at the thermocouple locations (units in mm): (**a**) TC1; (**b**) TC2; (**c**) TC3; (**d**) TC4, showing the weld bead geometry and the thermocouple position within each cross section.

Subsequently, a detailed cross-sectional analysis was conducted to determine the experimental dimensions of the weld seam and the thermocouple contact locations. The characteristic dimensions VW_V, HW_L and the weld penetration depth were measured for each thermocouple section, enabling the identification of the geometric parameters of the volumetric heat source model, named *b* and *c*. In addition, the spatial X and Y coordinates of TC1 (−0.47, −3.21), TC2 (−0.95, −2.50), TC3 (14.00, 0), and TC4 (−0.83, −3.30)—defined with respect to the origin—were determined, together with the thermocouple minimum contact distance to the fusion zone (MDFZ). These measurements are illustrated in Figure 16a–d.

**Figure 17:**
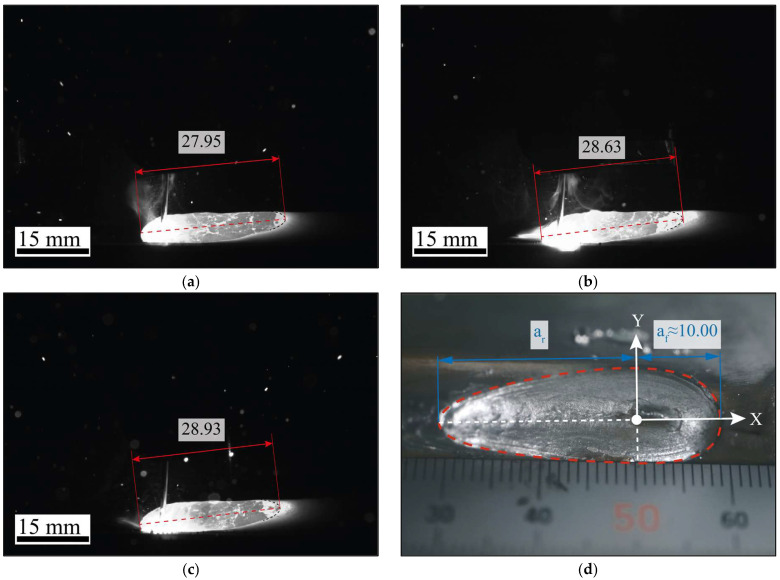
Experimentally measured longitudinal weld bead geometry (units in mm) acquired with a high-speed ACS-1 camera at different welding times: (**a**) 3 min; (**b**) 6 min; (**c**) 8 min; (**d**) end of the process.

Likewise, the longitudinal dimensions of the weld pool were experimentally determined using an ACS-1 high-speed camera, allowing the identification of the front and rear lengths of semi-major axes, ($a_{f}$ and $a_{r}$, respectively) of the volumetric heat source model under actual welding conditions. Figure 17a–c shows the evolution of the total weld pool length at different time instants during the process, while Figure 17d presents the average front length of the heat source.

**Figure 18:**
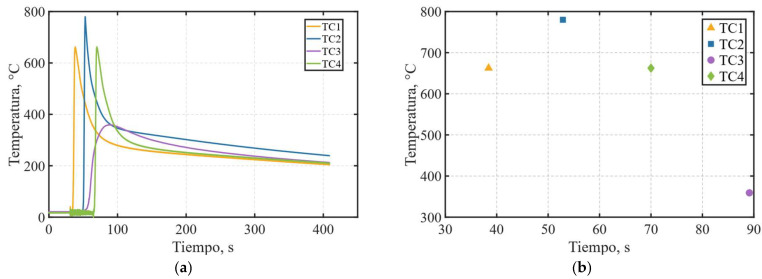
Experimental thermocouple temperature results (units in °C): (**a**) temperature–time histories recorded at all thermocouple locations; (**b**) corresponding peak temperatures measured during the welding process.

Finally, the temperature profiles recorded by the thermocouples illustrate the thermal response of the base material during the welding process, as seen in Figure 18a, characterized by a sharp temperature rise as the torch passes over the embedded thermocouples, followed by a more gradual decrease associated with heat dissipation through conduction and convection as the torch moves away. A higher peak temperature is observed at thermocouple TC2, as shown in Figure 18b, which is attributed to its closer proximity to the fusion zone tolerances previously shown in Figure 16b.

#### 3.1.2. Structural Response—Experimental

**Figure 19:**
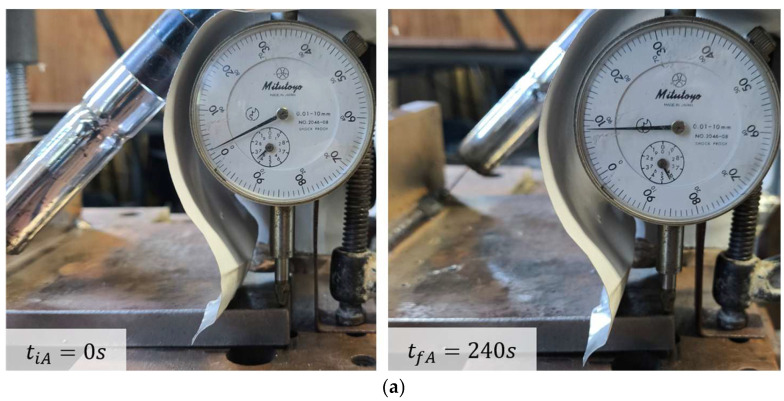

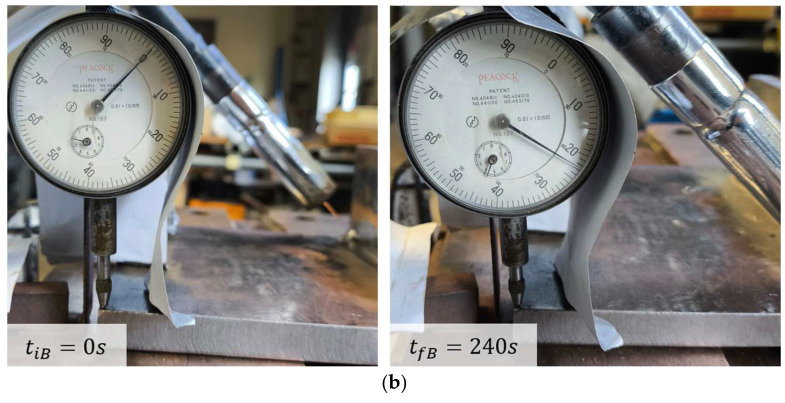
Dial indicator displacement readings (units in mm) before (**left**) and after (**right**) the welding process at: (**a**) point A; (**b**) point B.

The experimental displacements at points A and B were determined from the difference between the initial and final readings of Mitutoyo No. 2046-08 dial indicator (Mitutoyo Corporation, Kawasaki, Japan) and Peacock No. 107 dial indicator (OZAKI MFG. Co., Ltd., Tokyo, Japan) dial indicators which have a resolution of 0.01 mm and an accuracy of ±15 μm and ±20 μm respectively. The final readings were recorded 240 s after arc extinction, as no additional displacement of the base metal was observed beyond this time. The corresponding initial and final indicator readings for points A and B are shown in Figure 19a and Figure 19b, respectively.

**Figure 20:**
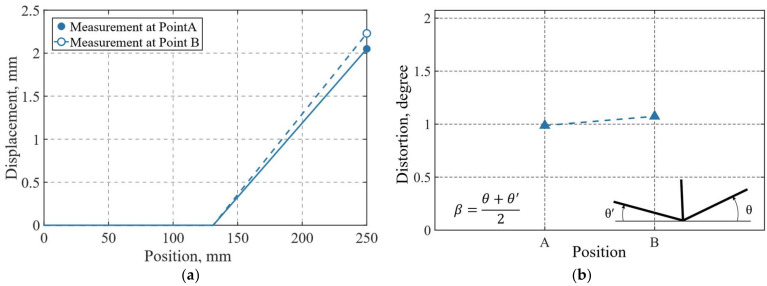
Experimental structural response after recorded 4 min after welding process completion: (**a**) measured displacement (mm); (**b**) measured angular distortion (degrees).

The maximum experimental displacements at points A and B were 2.05 mm and 2.23 mm, respectively, as shown in Figure 20a, while the angular distortion is presented in Figure 20b. These deformations are attributed to the temperature gradients generated during the welding process, which induce expansions and contractions in the material, primarily in the regions adjacent to the weld pool. A complete restraint condition was imposed on the specimen up to a length of 119 mm from its left end, corresponding to the fixation of the material in the experimental setup.

### 3.2. Simulation Study

#### 3.2.1. Thermal Response—Simulation

A preliminary numerical study was performed to evaluate the influence of the dimensionless heat source constant β and process efficiency on the simulation results. While both parameters aim to minimize the deviation between predicted and measured thermal histories, β exerts a more pronounced influence on the fusion zone morphology by governing the heat flux density. Accordingly, this parameter was iteratively adjusted to achieve geometric congruence between the simulated 1500 °C isotherm and the experimental fusion zone boundary. In turn, the process efficiency was evaluated from 75% to 100% in 5% increments, focusing on the 75–85% range documented in the literature for a GMAW process [43], and finally selected based on the highest correlation with measured peak temperatures and overall thermal histories.

**Figure 21:**
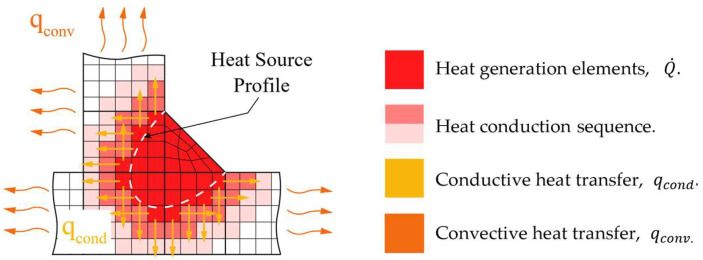
Thermal boundary conditions applied in the GMAW finite element model, including convective heat losses on all external surfaces and internal heat generation associated with the moving double-ellipsoidal heat source.

For this purpose, a β value of 2.5 was adopted, obtained from the calculation of the coefficient in each direction under the assumption that the power density falls to 0.08 of the maximum power in the surface of the heat source. In addition, a GMAW process efficiency value of 85% was selected. These values provided the best overall agreement in the predicted thermal behavior, while the remaining heat source parameters were determined directly from experimental measurements. Consequently, Figure 21 presents a schematic of the thermal boundary conditions implemented in the simulation, in which convection over all external surfaces is accounted for, along with heat generation within the elements associated with the double-ellipsoidal moving heat source formulation.

The influence of mesh sensitivity on the numerical results was assessed through a convergence test, which assumes that a mesh is optimal for the analysis when further refinement does not produce significant changes in the solution. The monitored response variables used to construct the convergence plot were the maximum temperature in the domain Tₘₐₓ and the temperature at the horizontal base of the weld bead Tₐ, as well as the simulation time corresponding to each iteration. The parameters employed in the subroutine to generate the convergence plot and the subsequent thermal analysis are summarized in Table 4.

**Figure 22:**
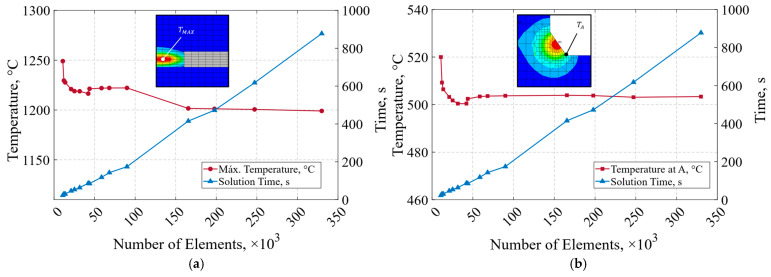
Convergence plot for mesh quality assessment: (**a**) maximum temperature; (**b**) temperature at the horizontal toe of the weld bead.

From both convergence plots (Figure 22a,b), it can be concluded that a mesh ranging between 25,000 and 58,000 elements provides optimal performance as it exhibits minimal variability relative to the convergence results obtained with finer meshes, showing only a 2% deviation in maximum temperature and 0.5% at point A. Additionally, this mesh density (25,000 elements), yields a significantly reduced computational cost, with an average simulation time of 82 s for the first 15 load steps of analysis.

**Figure 23:**
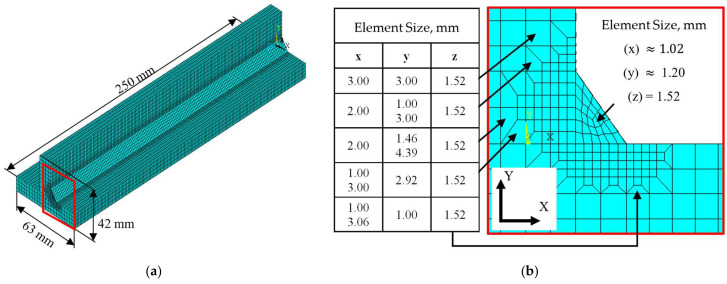
Finite element discretization of the numerical model: (**a**) isometric view of the full finite element mesh; (**b**) local view highlighting the element size and mesh density.

The resulting mesh layout and the corresponding element sizes used in the thermal and structural model are shown in Figure 23a and Figure 23b, respectively.

**Figure 24:**
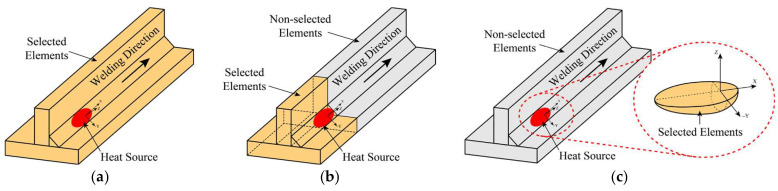
Representative element sets selected for moving heat source formulation under different selection strategies: (**a**) conventional full-domain selection; (**b**) partial selection limited to elements behind the heat source; (**c**) volume-based selection restricted to the weld seam volume.

Before final temperature field validation, a thermal simulation based on the proposed volume element selection strategy, Figure 24c, was performed and compared with a conventional approach and an alternative simulation time-reduction technique, referred to as the partial selection method. The main difference among these strategies lies in the nodal domain considered for applying the heat source formulation. While the conventional approach scans the entire element set of the model—including all nodes associated with both the beam geometry and the weld seam volume—to apply the corresponding heat generation, as illustrated in Figure 24a, the partial selection method restricts the nodal search to beam and weld seam elements located behind the moving heat source, as shown in Figure 24b.

**Figure 25:**
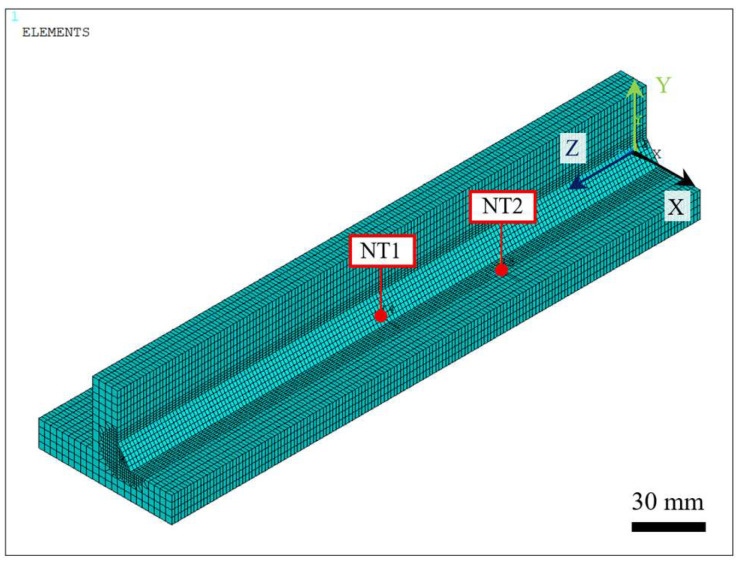
Representative monitoring locations for nodal temperature history used in the time comparison of element selection methods for moving heat source equation application.

Since the heat source application volume may significantly influence heat transfer within the model, the thermal results were also compared by evaluating the temperature response obtained using each element selection strategy. Accordingly, nodal temperatures were monitored at two representative locations: one located near the fusion zone and another at a superficial node. The first location (NT1) was recorded at coordinates (5 mm, −2.5 mm, 125 mm), while the second (NT2) was obtained at coordinates (17 mm, 0 mm, 80 mm) with respect to the global coordinate system, as illustrated in Figure 25.

The comparisons were conducted under identical numerical and computational conditions, and the comparison variable was defined as the total analysis time, corresponding to the macro execution time required to complete both the heating and cooling cycles, based on a single runtime measurement per method.

All simulations were performed on the same platform: a 64-bit Intel^®^ Core™ i5-8300H CPU, 32 GB of RAM operating at 2666 MHz, and an NVIDIA GTX 1050 Ti GPU device. For solver settings, the thermal simulation was made using a full solution scheme (ANTYPE,4; TRNOPT,FULL) with full Newton–Raphson nonlinear convergence (NROPT,FULL) and a consistent mass matrix formulation (LUMPM,OFF) to accurately capture steep thermal gradients. Time integration was governed by the Backward Euler method (TINTP,,,,1). Fixed time stepping was adopted to ensure numerical stability, disabling automatic stepping (AUTOTS,OFF) and applying controlled substeps during heating (NSUBST,5,,,1; KBC,0) and predefined increments during cooling (NSUBST,SUBVALUES,0,0).

**Figure 26:**
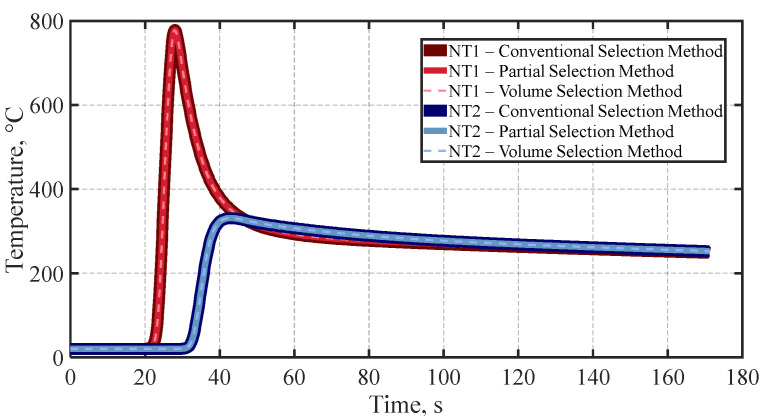
Temperature histories at two selected nodal locations—near the fusion zone (NT1) and at a distant surface location (NT2)—during the heating and cooling cycles, used for total wall-time comparison of the three element selection strategies.

Accordingly, the total wall-clock time was computed for the full simulation (heating plus cooling cycles) for conventional, partial, and volume selection methods, resulting in 0.7988, 0.5613, and 0.4061 h, respectively. In addition, negligible nodal temperature variation was observed at the monitored location for all strategies, as illustrated in Figure 25. As observed in Figure 26, the lines of temperature are overlapped because the results were almost the same.

**Figure 27:**
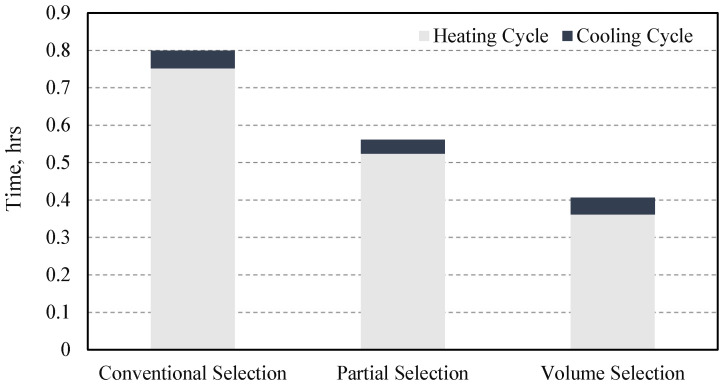
Overall computation time comparison between three elements selection strategies for heat source equation application over domain.

More representative results are presented in Figure 27, where the total simulation time is decomposed into the individual solution times for the heating and cooling cycles. As expected, the cooling cycle solution time exhibits negligible variation among the different selection methods due to identical simulation settings. In contrast, noticeable differences are observed during the heating cycle, where the solution time increases with the size of the element selection range. The maximum heating solution time of 0.7516 h. corresponds to the conventional selection method, while the minimum solution time of 0.3616 h. is obtained by implementing the volume selection method, thus contrasting a near 50% simulation time cost reduction in the volume element selection method without loss of accuracy.

**Figure 28:**
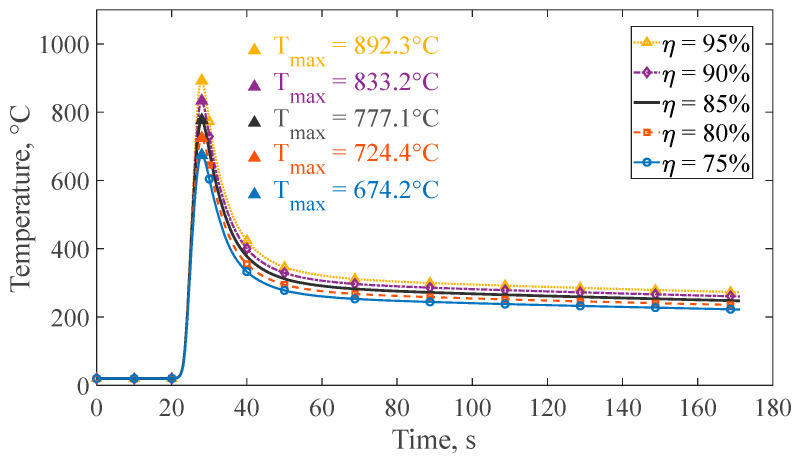
Temperature histories at node NT1 for different process efficiency values (75–95%).

Following, a sensitivity analysis was conducted to evaluate the influence of process efficiency on thermal predictions using the low-computational-cost volume element strategy. Simulation parameters were maintained according to Table 4, while efficiency was varied by ±5% and ±10% relative to the 85% selected value. For each test, thermal profiles were monitored at a representative node (NT1) located near the fusion zone at coordinates (5 mm, −2.5 mm, 125 mm), as previously illustrated in Figure 25. As expected, variations in process efficiency directly affect the predicted peak temperature due to the corresponding increase or decrease in the effective heat input supplied by the electric arc. Relative to the selected 85% efficiency case (peak temperature of 777.1 °C), a maximum deviation of +115.2 °C was obtained for a +10% efficiency variation, whereas a minimum deviation of −52.7 °C was observed for a −5% variation, as illustrated in Figure 28. These results show a proportional relationship between process efficiency and peak temperature, with a minimum of ±5% efficiency variations leading to peak temperature deviations below 7%, without altering the overall thermal response trend. The corresponding mechanical response derived from the maximum and minimum efficiency values of 95% and 75% will be addressed in the following section.

**Figure 29:**
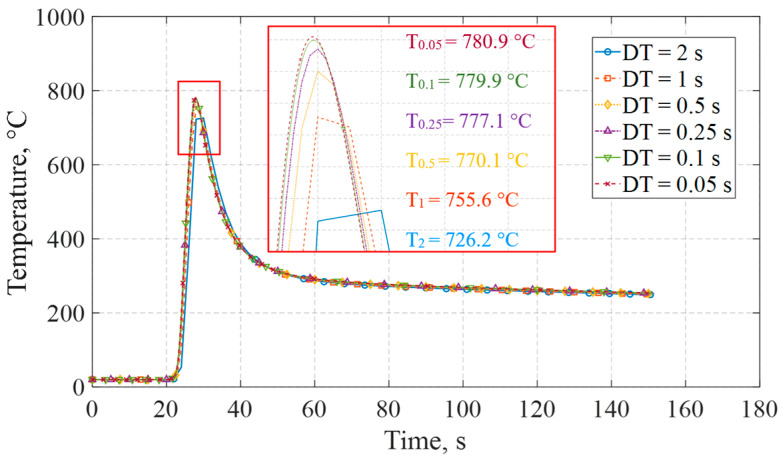
Temperature histories at node NT1 (5 mm, −2.5 mm, 125 mm) for different time-step (DT) values 0.05, 0.1, 0.25, 0.5, 1.0, 2.0.

Furthermore, the same simulation parameters used in the preceding analysis, along with the selected 85% efficiency value, were employed to perform a time-step (DT) sensitivity analysis of the thermal response. Time increments of 0.05, 0.1, 0.25, 0.5, 1.0, and 2.0 s were considered to capture both fine and coarse temporal behavior. As shown in Figure 29, taking the most refined solution (DT = 0.05 s) as numerical reference, peak temperature variations decrease consistently with time-step refinement, reaching 0.49% at DT = 0.25 s and 0.13% at DT = 0.1 s. Since smaller time steps only increase computational cost with negligible improvement in accuracy, whereas larger time steps significantly reduce the resolution of the thermal response, time increments of 0.25 and 0.5 s were adopted in the macro for the heating and cooling cycles, respectively, ensuring a balanced compromise between computational efficiency and accuracy. Likewise, since the mechanical distortion response is governed by the thermal field evolution, no meaningful variation in structural results is present within the 0.05–0.5 s increment range.

**Figure 30:**
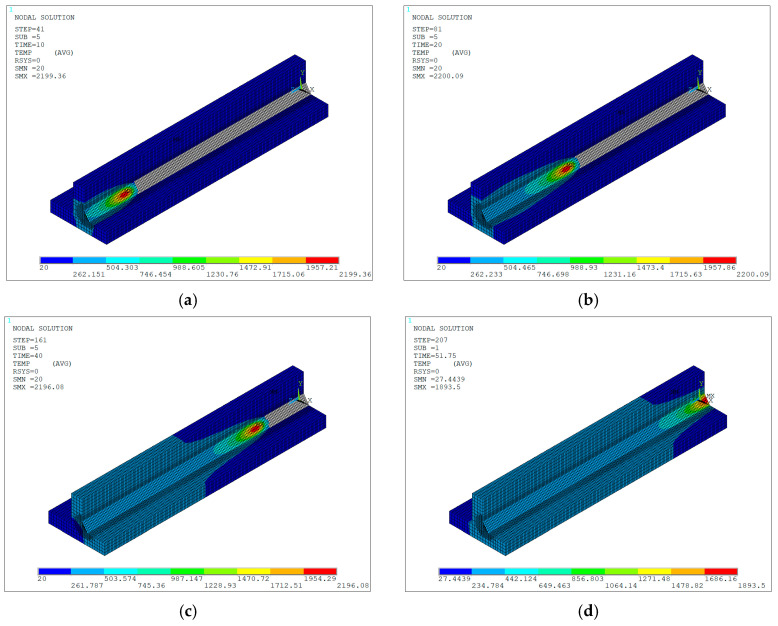
Isometric view of numerically predicted temperature contours (units in °C) during the heating cycle at different time instants: (**a**) 10 s; (**b**) 20 s; (**c**) 40 s; (**d**) 51.75 s.

Finally, the temperature distribution during the welding process was obtained, thereby generating the temperature contours in the specimen throughout the heating cycle for different time instant, as shown in Figure 30a–d. These contours reveal the evolution of temperature as time progresses and the heat input from the active electric arc increases on the materials. The gray-colored elements in the weld bead are deactivated or death elements and have no influence on the solution of the current load step, whereas elements inside the heat source at a temperature equal to or exceeding the JIS-SM490A steel melting point (1500 °C), represent the liquid material of the weld pool.

**Figure 31:**
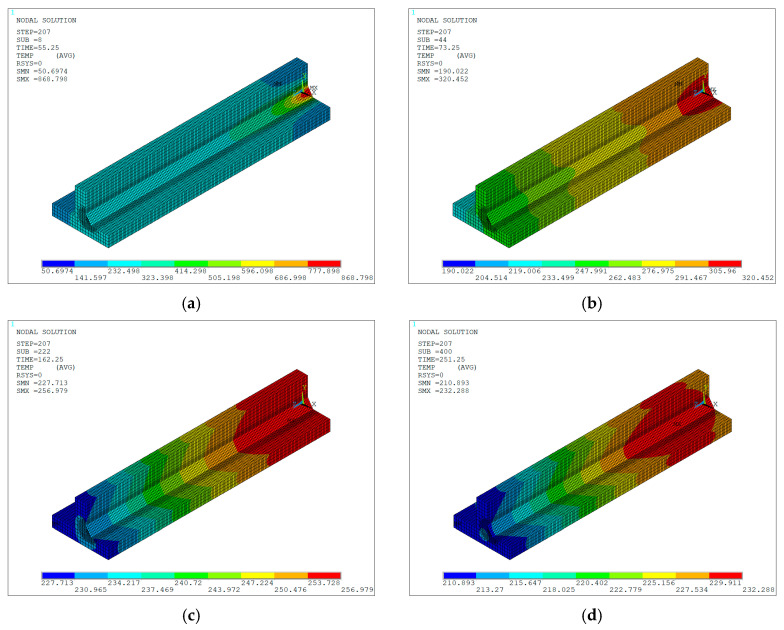
Numerically predicted temperature contours (units in °C) during the cooling cycle at different time instants: (**a**) 55.25 s; (**b**) 73.25 s; (**c**) 162.25 s; (**d**) 251.25 s.

Once the heat source was deactivated, the temperature distribution corresponding to the cooling cycle was obtained, represented by the thermal contours of the specimen for different time instants, as shown in Figure 31a–d. In this stage, a progressive decrease in temperature is observed, beginning in the region closest to the point where the torch movement ended—where the highest values are retained—and extending toward the more distant areas. As time is running out, the temperature gradually spreads throughout the remaining volume and, given sufficient cooling time, ultimately reaches a state of thermal equilibrium across the entire domain.

**Figure 32:**
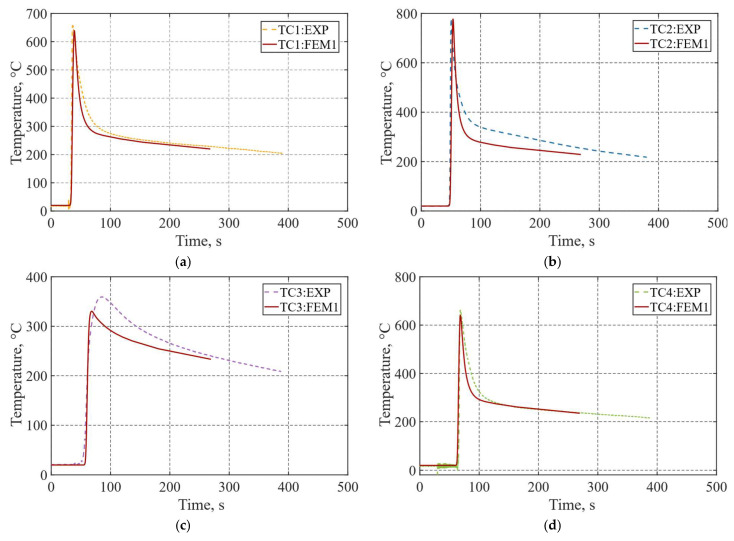
Comparison of experimental and numerical temperature histories (°C) at the thermocouple locations: (**a**) TC1; (**b**) TC2; (**c**) TC3; (**d**) TC4.

Figure 32a–d presents a comparison between the temperature histories recorded by the thermocouples and those obtained numerically from the simulation. As previously shown, thermocouple location uncertainty was accounted for through a macroscopic analysis, and the post-manufacturing measured positions were incorporated into the simulation to consider the actual measurement point deviations. The measured cooling gradients show almost similar curves to those predicted by the finite element model. The highest numerical peak temperature was recorded at TC4, located closest to the fusion zone (FZ), reaching 777.03 °C. This value is in close agreement with the corresponding experimental maximum of 779.96 °C, resulting in a difference of 2.93 °C. Despite the model simplifications, the temperature history differences remain limited, with average absolute deviations of 18.48 °C, 42.94 °C, and 11.60 °C for TC1, TC2, and TC4, respectively. For TC3, positioned farther from the fusion zone, both numerical and experimental cooling curves exhibit similar behavior; however, a noticeable difference is observed, which can be attributed to the simplified heat loss assumptions, particularly radiation and convection. These simplifications may lead to a less aggressive cooling rate, resulting in a smoother temperature decay and a reduced slope compared to the experimental curve. Nevertheless, a relatively low overall deviation was obtained, with a maximum value of 26.88 °C. Generally, the developed simulation model demonstrates good agreement with experimental data.

#### 3.2.2. Structural Response—Simulation

**Figure 33:**
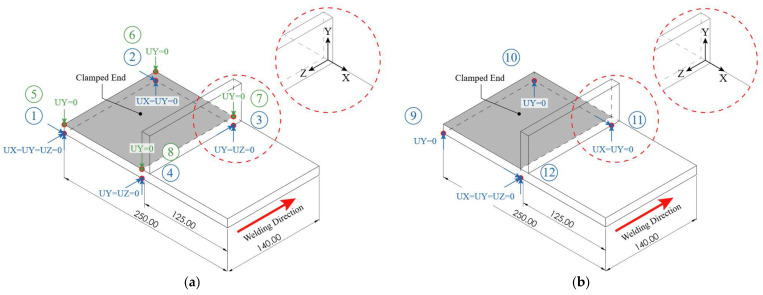
Structural simulation boundary condition setups: (**a**) eight-node lower and upper clamping configuration; (**b**) simplified four-node lower clamping configuration.

To represent the physical clamping conditions of the structural test, displacement at eight nodes located at the fixed section, corresponding to the four lower (blue-colored DOFs) and four upper (green-colored DOFs) corners of the clamped end, were constrained, as illustrated in Figure 33a. To evaluate the sensitivity of the structural response to clamping conditions, a second, less-constrained configuration was imposed by restricting displacement at four nodes located on the lower face of the clamped end, as shown in Figure 33b.

Both configurations varied the nodal locations near and far from the welding area according to the beam geometry parameters W1 and T2, corresponding to the bottom flange width and web thickness, respectively, taking values of −125 mm (−W1/2) for nodes far from the welding zone (locations 1, 2, 5, 6, 9 and 10) and −6 mm (−T2/2) for those near it (locations 3, 4, 7, 8, 11 and 12) in the *X* direction. The lower and upper positions were controlled by the bottom flange thickness T1, located at −12 mm (−T1) and 0 mm, respectively, in the *Y* direction, according to the defined coordinate origin.

A single analysis was conducted for each case using the eight-node and simplified four-node clamping configurations and evaluated through the full heating stage followed by 240 s of cooling. The resulting UY displacement contour plots were then compared for analysis.

**Figure 34:**
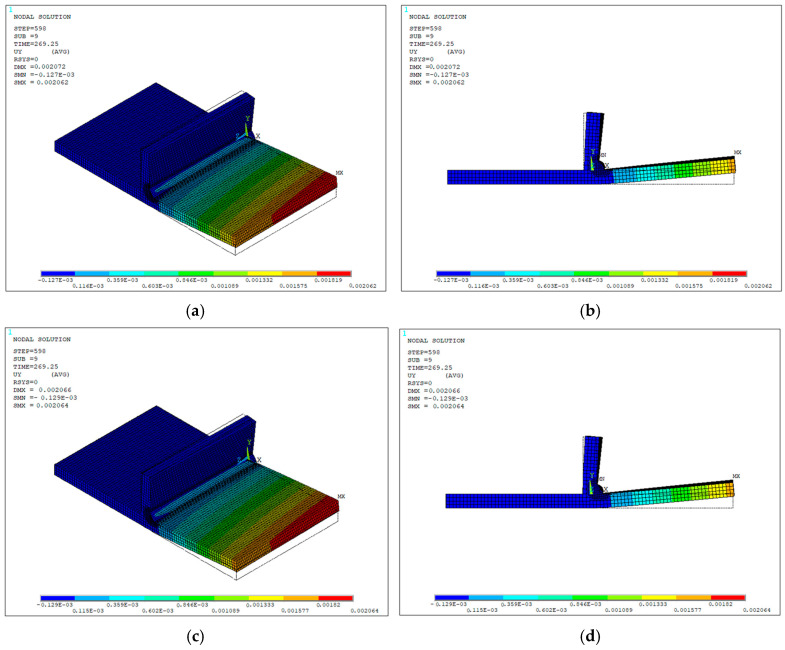
Vertical displacement contours UY (in meters) at the final step of the structural analysis for the clamping configuration comparison: (**a**) isometric view, 8-node configuration; (**b**) frontal view, 8-node configuration; (**c**) isometric view, 4-node configuration; (**d**) frontal view, 4-node configuration.

The simulation parameters were kept consistent with those shown in Table 4, modifying only the beam geometry parameters corresponding now to the structural specimen dimensions. For solver settings, the mechanical simulation was made using a nonlinear static structural scheme (ANTYPE,4) with a full Newton–Raphson linear convergence (NROPT,FULL). Geometric nonlinearity was included (NLGEOM,ON) to account for large-deflection effects. Convergence was enhanced through the activation of the line search algorithm (LNSRCH,ON) and automatic load stepping (AUTOTS,ON) with controlled substeps definition (NSUBST,3,12,1; KBC,0). The reference temperature was set to ambient conditions (TREF,T_INF) and a negligible thermal expansion coefficient above 1500 °C was employed to avoid unrealistically large thermal expansions. Since both clamping approaches aim to represent the physical fixation of the constrained area, negligible variations were found in the structural response between the eight-node and four-node clamping configurations, with maximum UY displacements at the final step of 2.062 mm and 2.064 mm, respectively, as illustrated in Figure 34a–d. It is important to note that the defined constraints and restricted DOFs directly influence the structural response; therefore, simplifications during the setup stage must be performed as close as possible to the experimental clamping conditions to improve results accuracy.

**Figure 35:**
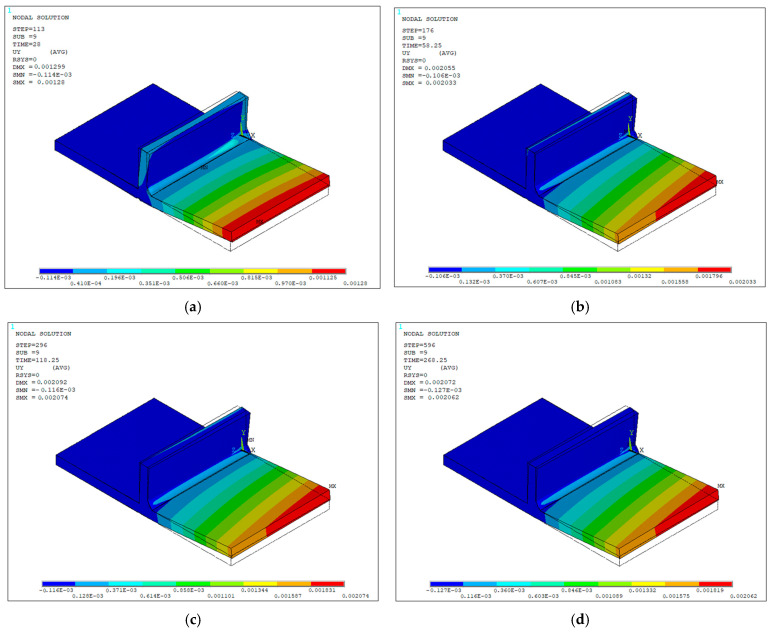
Vertical displacement contours UY (units in meters) at different time instants during the structural analysis: (**a**) 28 s; (**b**) 58.25 s; (**c**) 118.25 s; (**d**) 268.25 s.

The final structural response was obtained using the selected parameter values: 85% process efficiency, time steps of 0.25 and 0.5 s for the heating and cooling stages, respectively, and the eight-node clamping configuration. Figure 35a–d show the UY displacement contours at different time instants during the cooling cycle, which begins 28 s after the start of the process. The largest displacements are concentrated at the free end of the specimen, and they are attributed to the boundary condition imposed at the opposite fixed end, as well as being consistent with the temperature gradients generated, where expansion in the weld bead during heating and its subsequent contraction during cooling induce angular distortion in the base metal at the free end.

**Figure 36:**
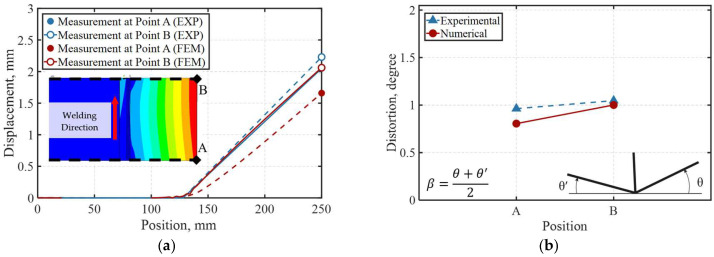
Comparison of experimental and numerical structural response after welding: (**a**) measured vertical displacement (mm); (**b**) measured angular distortion (degrees).

From the vertical displacement contours (UY), nodal displacement values were extracted at the final load step of simulation along lines A and B by selecting all surface nodes corresponding to each reference line. As previously stated, the maximum deformations occurred at the free ends of the flange at both measurement points, reaching 1.66 mm at point A and 2.07 mm at point B. This behavior is attributed to the moving heat source, the welding direction, and the thermal expansion followed by contraction of the base material during the heating and cooling of the weld seam. As the heat source advances, the angular distortion increases with distance from the weld start, which is reflected in larger displacement contours toward end B and a more pronounced angular deformation near line B. Figure 36a,b compare numerical and experimental displacement and angular distortion results. Good agreement is observed, with displacement differences at the free end and measurement points A and B limited to 0.16 mm and 0.39 mm, respectively, and an angular distortion deviation below 1°, indicating that the simulation reliably captures the experimentally observed deformation trends.

Finally, to assess the impact of process efficiency variations on the structural response, the efficiency sensitivity analysis previously conducted for the thermal response was extended by evaluating the corresponding distortion associated with the maximum temperature deviations obtained at the lowest and at the highest efficiency values (75% and 95%). As in the previous analysis, the simulation parameters were kept consistent with those defined earlier, employing the eight-node clamping configuration.

**Figure 37:**
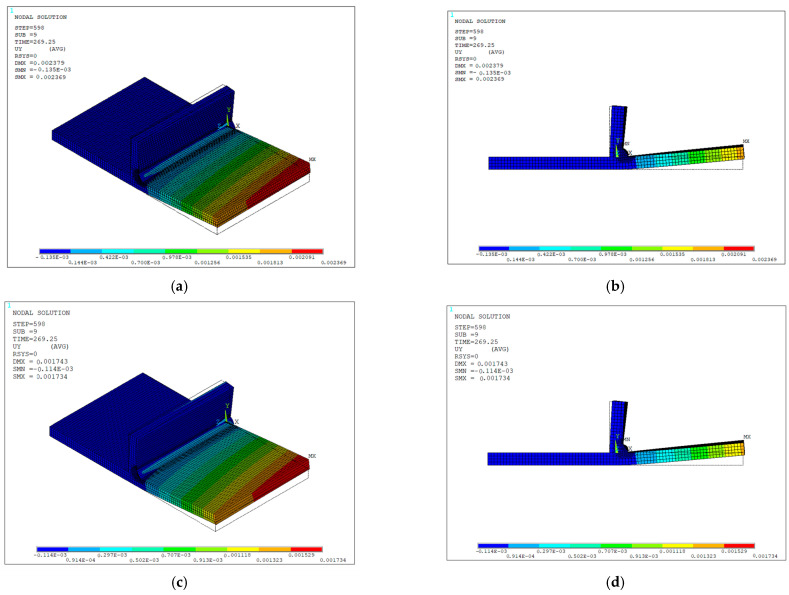
Vertical displacement contours UY (units in meters) at the final step of the structural analysis for process efficiency variations: (**a**) isometric view, η=95%; (**b**) frontal view, η=95%; (**c**) isometric view, η=75%; (**d**) frontal view, η=75%.

Consistent with the temperature trend, the maximum distortion increases to 2.36 mm at 95% efficiency, as shown in Figure 37a,b, and decreases to 1.73 mm at 75%, as illustrated in Figure 37c,d. Relative to the 2.07 mm maximum distortion at line B for 85% efficiency, a +14% deviation occurs at 95% efficiency and a −16.43% deviation at 75% efficiency. These results indicate a distortion sensitivity range of −0.34 mm to 0.29 mm for ±10% efficiency variations, highlighting that the structural response relies on the thermal calibration process, with process efficiency playing a significant role through its influence on peak temperatures.

## 4. Conclusions

A three-dimensional thermo-mechanical APDL macro for GMAW analysis is presented, implementing an automated and computationally efficient methodology for different T-joint configurations. The proposed approach enables the direct generation of complete finite element models from user-defined parameters, including base-material geometry, weld bead dimensions, welding conditions, and heat-source characteristics, eliminating the need for manual model construction or interactive preprocessing within the FEM environment.

The development of the code in the parametric language in ANSYS allows a fast and accurate prediction of temperature distribution and distortion, which allows for better and affordable decision making with respect to the design of flanged structures and welding sequence.

A volume element selection strategy aiming to reduce simulation time for this type of analysis was successfully implemented in the macro, achieving a significant reduction in solution time by up to 50% without compromising analysis accuracy.

The agreement between the numerical predictions and the experimental temperature histories, with a minimum variation of 11.6 °C observed at thermocouple four, together with the displacement field results—showing a maximum displacement difference at the free-end point B of 0.22 mm—confirms the correct implementation and functionality of the automated procedure. Overall, the results demonstrate that the proposed macro constitutes a useful tool to support thermo-mechanical welding analyses of T-joints, significantly simplifying model setup while preserving the capability to evaluate different joint geometries and welding conditions through parametric input and a moving volumetric heat source model.

Nevertheless, certain limitations should be acknowledged. The model relies on the availability of temperature-dependent thermal and mechanical properties and does not consider phase transformations; therefore, microstructural evolution cannot be predicted. While the proposed framework demonstrates high efficiency and accuracy for the SM490A steel T-joints studied, its application to other materials or geometries significantly different from those analyzed and should be interpreted within the context of the adopted model simplifications.

Finally, due to the automated and parametric nature of the macro, future work will aim to enhance its robustness for a wider range of T-joint geometries and configurations, as well as to extend its applicability to more complex welding procedures, including multi-pass or double-bead welds.

## Figures and Tables

**Table 1:** Comparison of key parameters of prior GMAW T-joint studies versus present work.

Reference	End-to-End Automation	Parametric Geometry	Heat Source Model	Time Efficiency Strategy	Primary Limitation
Musolino et al., 2025 [25]	No	Partial	Goldak	Adaptive mesh refinement	High setup time per geometry
Liu et al., 2025 [26]	Partial	Yes	Goldak	Local thermo-mechanical analysis and local–global strain mapping	High setup time per analysis
Wang y Qian, 2024 [31]	No	Partial	Goldak	Automated heat source calibration	Extensive data set for training
Wang et al., 2023 [29]	Partial	Partial	Goldak	Step-by-step inherent strain loading	Dependence on high-cost calibration
Kollár, 2023 [30]	Partial	No	Goldak	Local mesh refinement and linear heat scaling	Non-parametric geometry
Wang et al., 2020 [27]	Partial	No	Goldak	Local mesh refinement	Non-parametric geometry
Perić et al. 2019 [28]	No	Partial	Simplified	Shell/3D coupled modeling	Simplified heat source model
Xu et al., 2013 [16]	Partial	Partial	Modified Goldak	Local mesh refinement	Specific thin plates
This work	Complete	Yes	Goldak	Volume-based element selection	Simplified boundary conditions

**Table 2:** Chemical composition of JIS-SM490A steel.

	C (%)	Si (%)	Mn (%)	P (%)	S (%)
JIS-SM490A	max. 0.22	max. 0.55	max. 1.6	0.035	0.035

**Table 3:** Welding conditions.

Current (W)	Voltage (V)	Welding Speed (mm/s)	Shielding Gas CO_2_ (l/min)	Electrode Stick Out (mm)	Tilt Angle (deg)
280	31.97	5	20	25	45

**Table 4:** Simulation parameters used for convergence study and the subsequent thermo-mechanical analysis.

Parameter	Value	Variable
Flange width (m)	63 × 10^−3^	W1
Web height (m)	42 × 10^−3^	W2
Flange thickness (m)	12 × 10^−3^	T1
Web thickness (m)	12 × 10^−3^	T2
Total length (m)	250 × 10^−3^	L
Weld bead horizontal leg (m)	6.125 × 10^−3^	HW_L
Weld bead vertical leg (m)	8.78 × 10^−3^	VW_L
Voltage (V)	31.97	VOLT
Current (A)	280	CURR
Welding speed (mm/s)	5	SPEED
Arc efficiency (%)	85	η
Dimensionless heat source constant	2.5	β
Ambient temperature (°C)	20	T_INF
Convective film coefficient (W/m^2^·°C)	25	h
Heat source half-width (m)	5.35 × 10^−3^	b
Heat source depth penetration (m)	7.88 × 10^−3^	c
Heat source front quadrant length (m)	10 × 10^−3^	$a_{f}$
Heat source rear quadrant length (m)	18.5 × 10^−3^	$a_{r}$
Heating cycle time increment (s)	0.25	V_TIMEINC
Cooling cycle time increment (s)	0.5	TIMESTEPS

## Data Availability

The original contributions presented in this study are included in the article/Supplementary Materials. Further inquiries can be directed to the corresponding author.

## Supplementary Materials

The following supporting information can be downloaded at: https://www.mdpi.com/article/10.3390/ma19051021/s1.
