# Supplementary material for: Automated and Low Computational Cost Thermo-Mechanical Simulation of Arbitrary GMAW T-Joint Welds Using a Moving Heat Source
Source: Materials (Basel). 2026 Mar 6;19(5):1021. doi: 10.3390/ma19051021 (PMC12985572; doi:10.3390/ma19051021)
Supplement: Supplementary file 1 [file materials-19-01021-s001.zip › materials-4134382-supplementary.pdf]

Benchmark case definition for the work:

Automated and Low Computational Cost Thermo-Mechanical Simulation of Arbitrary GMAW T-joint Welds Using a Moving Heat Source

1. Macro setup preparation and execution

a) Place all ".mac" files in a new folder on the desktop.
b) Create a new ANSYS APDL job by setting the newly created folder as the *Working Directory*. The *Job Name* must be defined as **"MobileHeatSource."**
c) In the command window, type "**A_MAINV17**" and execute the command. Four pop-up windows will appear to define the simulation conditions. All parameters are pre-defined; click **ACCEPT** in each window.
d) The simulation will start automatically.

2. Overall macro input parameters

The macro parameters used in the benchmarks were established based on the final structural analysis conducted in this study, following the configuration shown.

As a computational reference, this benchmark was conducted on a 64-bit Intel® Core™ i9-13980HX (24 threads processor), 32 GB of RAM operating at 5600 MT/s, and an NVIDIA RTX 4070 GPU device.

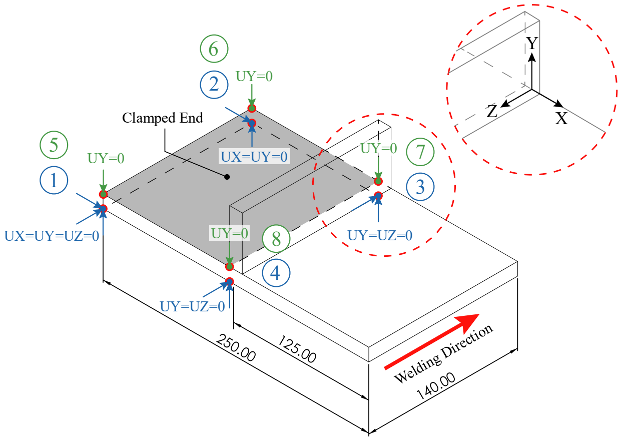

Main stimulation parameters are summarized below:

The macro is currently charged with the benchmark input values as default, no user adjustment needed.

Parameter	Value	Variable
Flange width (m)	250E-3	$W1$
Web height (m)	62E-3	$W2$
Flange thickness (m)	12E-3	$T1$
Web thickness (m)	12E-3	$T2$
Total length (m)	140E-3	L
Weld bead horizontal leg (m)	6.125E-3	HW_L
Weld bead vertical leg (m)	8.78E-3	VW_L
Voltage (V)	31.97	$VOLT$
Current (A)	280	$CURR$
Welding speed (mm/s)	5	$SPEED$
Arc efficiency (%)	85	η
Dimensionless heat source constant	2.5	β
Ambient temperature (°C)	20	T_INF
Convective film coefficient ($W/m^2\cdot°C$)	25	h
Heat source half-width (m)	5.35E-3	b
Heat source depth penetration (m)	7.88 E-3	c
Heat source front quadrant length (m)	10E-3	a_f
Heat source rear quadrant length (m)	18.5 E-3	a_r

Heating cycle time increment (s)	0.25	$V\_TIMEINC$
Cooling cycle time increment (s)	0.5	TIMESTEPS

3. Thermal response

As the thermal results export process is enabled, a temperature results file will be automatically generated at the end of the thermal analysis in the root working directory. The resulting file, *"TEMPERATURE_RESULTS.csv"*, contains the recorded temperatures at the specified nodal locations. Temperature histories such as those shown below are expected at this stage.

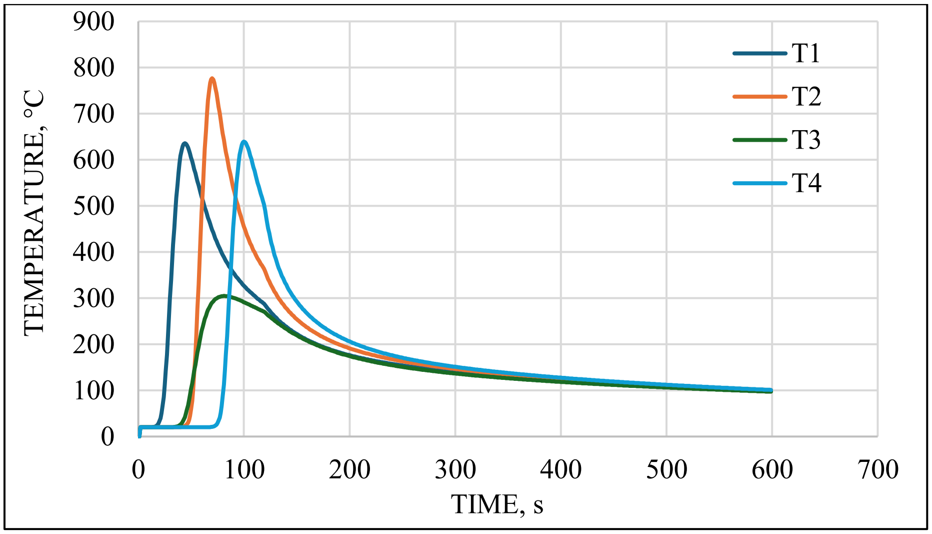

Heating cycle simulation time:
A4_TIMEWALL_THERMAL_HEATING =
0.191 hrs.

Full Heating & Cooling cycle simulation time:
A4_TIMEWALL_THERMAL_TOTAL =
0.258 hrs.

4. Structural response

Finally, as established in this study, the structural response at the selected time steps (28, 58.25, 118.25, and 268.25 s) is expected to be as follows:

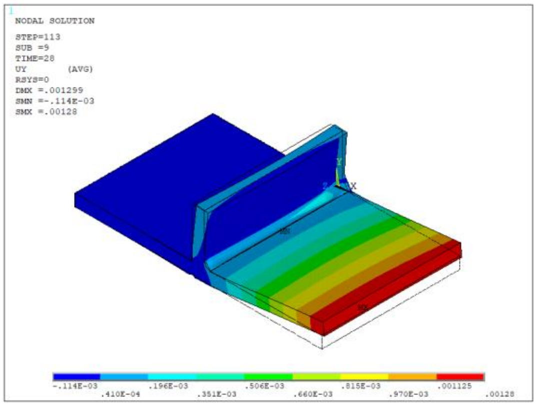

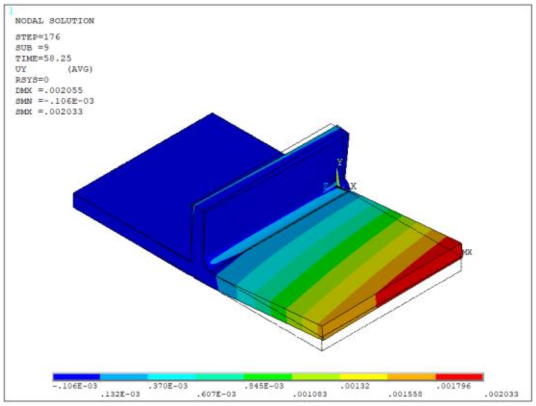

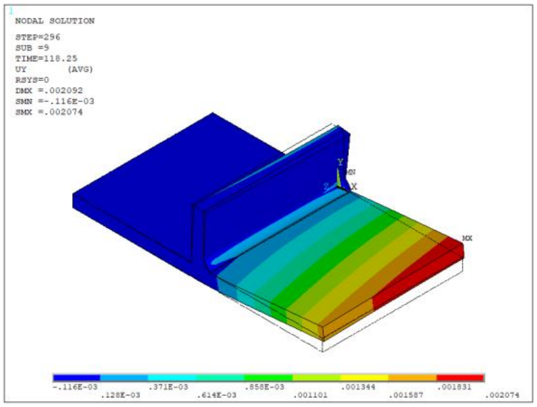

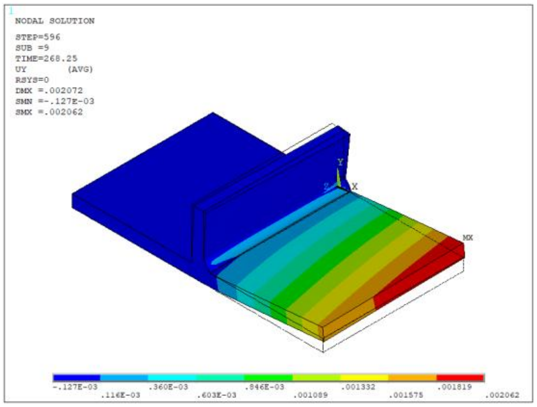

Structural simulation time:

A4_WALLTIME_STRUCTURAL = 9.22 hrs.
